# Communicating health and science to the public: a role for scientists and academic researchers

**DOI:** 10.5365/wpsar.2023.14.3.1079

**Published:** 2023-08-14

**Authors:** Jocelyne Marie Basseal, Mary-Louise McLaws, Sophie Scott, Sharon Salmon

**Affiliations:** a Sydney Infectious Diseases Institute, Faculty of Medicine and Health, The University of Sydney, Sydney, Australia.; b UNSW Medicine, School of Public Health and Community Medicine, University of New South Wales, Sydney, Australia.; c Medical School, The University of Notre Dame, Western Australia, Australia.; d World Health Organization Regional Office for the Western Pacific, Manila, Philippines.; e Indo-Pacific Centre for Health Security, Department of Foreign Affairs and Trade Australia, Canberra, Australia.

The coronavirus disease (COVID-19) pandemic identified valuable lessons for Australia’s public health response including the need for timely, clear and open communication to the public. (*1*) With the launch of the World Health Organization Western Pacific Region Communication for Health (C4H) initiative, (*2*) insights from social, behavioural and communication sciences contribute to improved health outcomes. Close collaboration between journalists and scientists is important, particularly during a pandemic, for developing trust in science. (*3*) This perspective piece highlights the importance of engaging trusted scientists and academic researchers during public health emergencies while ensuring they receive communication training to confidently interact with journalists and the public.

During the COVID-19 pandemic, science evolved rapidly and government decisions were constantly updated. However, they were often challenged by the public, for example, the effectiveness and side-effects of COVID-19 vaccines and the transmission route of SARS-CoV-2. The volume of information generated during the COVID-19 pandemic was addressed during the 73rd World Health Assembly, where Member States were urged to unite to manage the “infodemic,” and to combat and prevent the spread of mis- and disinformation while respecting freedom of expression. (*4*) Social media became an invaluable source of material for journalists, with clinicians, scientists and academic researchers posting facts and their observations using these channels. It was well documented that automated online accounts or software robots known as “bots” disproportionately contributed to controversial conversations online and influenced opinion trends, (*5*) and this was amplified during the pandemic with up to 66% of bots actively posting about COVID-19. (*6*) In addition, beliefs in misinformation were significantly associated with lower levels of digital health literacy, the perceived threat of COVID-19, confidence in government and trust in scientific institutions. (*7*)

The research community generated a large number of research studies on COVID-19, with publishers supporting open access and sharing resources to rapidly disseminate scientific information. (*8*) Commissioning research with trusted local researchers and the rapid creation of evidence from emergency response projects were successfully used to inform the public health response. (*9*) However, conducting research is not solely about contributing to the evidence base; equally important is communicating research findings to the target audience to achieve an effective public health response.

The COVID-19 pandemic introduced unique and fast-growing challenges for health communicators. Ratzan et al. (*10*) suggested three areas of capacity building: the need for proactive communicators to combat false information and establish trusted leadership; the importance of planning for unpredictability while acknowledging the uncertainty as scientific evidence evolves; and to remain people-centred with interventions for health and media literacy. The health literacy, language and cultural needs of a community should also be considered when developing public health messaging about COVID-19. (*11*)

Despite the infodemic, lack of trust in governments, rapidly evolving science and challenges in health communication, journalists still needed to meet daily reporting deadlines. As journalists play a critical role in influencing public opinion, they have a responsibility not to publish inaccurate or misleading headlines that cause fear and diminish countermeasures against the outbreak. (*12*) Addressing these issues resulted in the media shifting towards the use of scientists (*13*) and academic researchers as spokespersons, with virologists, infectious disease specialists and epidemiologists most commonly engaging with them. These scientists and academic researchers were able to provide interpretations of new research for the public and became crucial to the public’s understanding of COVID-19.

At a tumultuous time during a global pandemic when a “war of words” (*14*) can misguide the public, there is a need to turn to credible sources of information from experts. As an example, Australia turned to several epidemiologists and infectious disease specialists for balanced, honest, authentic and evidence-based advice, scaling up the engagement of scientists and academic researchers with the media became increasingly evident. It is critical for scientists and academic researchers to further develop their science communication skills and to be confident when collaborating with journalists as the media continually seeks experts for commentary. Building strong relationships with journalists may help combat misinformation and misconceptions of science and research and might reinforce important messages from government-funded public health campaigns. Considering the insights gained from the COVID-19 pandemic, it is time to prioritize and invest in science communication training and build capacity for scientists and academic researchers to engage with the media. Equipping infectious disease experts, virologists, epidemiologists and many other academic researchers with effective public engagement and science communication skills may enable them to become influential champions in rebuilding trust in science during future disease outbreaks.
